# Modulation of insulin aggregation by betaine and proline directly observed via real‐time super‐resolution microscopy

**DOI:** 10.1002/pro.70149

**Published:** 2025-05-15

**Authors:** Steen W. B. Bender, Jacob Kæstel‐Hansen, Vito Foderà, Nikos S. Hatzakis, Min Zhang

**Affiliations:** ^1^ Department of Chemistry University of Copenhagen Copenhagen Denmark; ^2^ Center for Optimized Oligo Escape and Control of Disease University of Copenhagen Copenhagen Denmark; ^3^ Center for 4D Cellular Dynamics University of Copenhagen Copenhagen Denmark; ^4^ Department of Pharmacy University of Copenhagen Copenhagen Denmark

**Keywords:** modulation, protein aggregation, real‐time direct observation, REPLOM, super‐resolution microscopy

## Abstract

Protein aggregation is associated with a spectrum of neurodegenerative diseases. Although many small ligands have been found to modulate or inhibit protein aggregation, their molecular mechanisms remain unclear. One reason for this is the inherent heterogeneity of protein aggregation pathways with different kinetics that result in the coexistence of multiple structures, for example, protein spherulites and fibrils, challenging the analysis of protein–ligand interactions. To address this issue, we evaluated the roles of betaine and proline in insulin aggregation. We employed our recently developed super‐resolution microscopy real‐time kinetics via binding and photobleaching localization microscopy (REPLOM) to directly observe the formation and morphological evolution of individual insulin aggregates in real time, with or without betaine/proline. Utilizing our machine learning approach, we monitor the effect of betaine and proline on the aggregation pathways and extract the growth kinetics of each individual aggregate type. Our results show that a high concentration of betaine or proline modulates the heterogeneity of the final aggregates, leading to the formation of smaller aggregates in a mixture with spherulites. The fraction of small aggregates increases with betaine/proline concentration, highlighting the heterogeneity of protein aggregation, and our toolbox can unravel the effects of small molecule ligands on individual protein aggregation pathways and the resulting aggregate types and abundances.

## 1 INTRODUCTION

Protein misfolding and self‐assembly into amyloid aggregates have been implicated in a wide range of diseases, including, among others, Alzheimer's, Parkinson's, and type II diabetes (Chiti & Dobson, 2006, 2017). Beyond healthcare, protein aggregation reduces the yield of protein expression and purification, affecting the formulation and price of protein‐containing medications (Cromwell et al., 2006). In addition, in dairy processing plants, protein aggregates may clog pipes and reduce productivity (Law & Leaver, 2000). As a result, it is important to find effective ligands that can modulate protein aggregation.

Despite extensive efforts to modulate protein fibrillation and the successful implementation of chaperones and osmolytes (Nagaraj et al., 2022; Venkatraman et al., 2020), the current understanding of the modulation mechanisms primarily relies on ensemble methods, for example, bulk fluorescence‐based techniques, which average the behavior and often provide a single explanation of the process (Hervás & Oroz, 2020; Wentink et al., 2020). However, protein aggregation is difficult to describe with a single descriptor because it is inherently heterogeneous: besides fibrils, additional species like spherulites and particulates can co‐exist in a solution (Krebs et al., 2009). These aggregate species originate from various self‐assembly pathways, morphological evolution, and kinetics, and are as important as fibrils. For instance, spherulites are abundant both in vitro and in vivo, such as in plaques from animal brains with disease equivalent to Alzheimer's disease and the new variant of Creutzfeldt‐Jakob disease (Exley et al., 2010; House et al., 2011). Interestingly, recent studies have provided new clues that the aggregate morphology can elicit diverse immune responses (Thorlaksen et al., 2023). Therefore, it is as important to modulate the formation of protein aggregate morphology as it is to control the protein fibrillation reaction. Consequently, understanding the modulatory effect of ligands at the single aggregate level is of great importance for the development of effective inhibitors/modulators targeting specific types of aggregates. Single‐molecule techniques, which dissect the mechanisms of complex processes without the associated ensemble averaging, could be a powerful tool for protein aggregation and modulation studies.

We recently developed a super‐resolution real‐time kinetics via binding and photobleaching localization microscopy (REPLOM) to directly observe the formation of human insulin spherulites in real time, decipher the heterogeneous growth pathways, and extract kinetics of individual spherulites for the first time (Zhang et al., 2022). Here we took advantage of the aggregation condition that favors insulin spherulite formation and extended the use of REPLOM to investigate how small ligands modulate insulin spherulite formation at the single aggregate level. Combined with the turbidity assay, which reports ensemble aggregation kinetics, and spinning disk confocal microscopy, which shows the final aggregate structures, we unraveled the modulatory effect of betaine and proline, known inhibitors of insulin fibrillation (Choudhary et al., 2015), on the formation of insulin spherulites. Our data showed that both betaine and proline require a relatively high concentration to effectively modulate the formation of insulin spherulites. This is consistent with previous findings that betaine and proline are generally effective only at relatively high concentrations (Arora et al., 2004; Gibson & Murphy, 2006). Although there is very little effect on the ensemble kinetics at low concentrations of betaine or proline samples, spinning disk images showed that the presence of a low concentration of betaine/proline could induce more small aggregates to form than in the absence of betaine/proline. The proportion of small aggregates increased with the concentration of betaine or proline. The automatic identification of individual aggregates observed by REPLOM was achieved by our machine learning framework segmentation and morphological fingerprinting (SEMORE) (Bender et al., 2024), with an accuracy of >90%. Then the corresponding growth kinetics and morphology development of each individual aggregate were extracted. We found that the small aggregates have relatively slower growth rates and observed that the aggregation processes of some small aggregates were terminated at a certain time point. Our work offers fresh perspectives on the modulatory effects of small ligands on protein aggregation. By combining spinning disk imaging and REPLOM, we successfully observed the influence of small ligands on the morphology and growth rate of protein aggregates at the single aggregate level. Our framework can potentially be used to design modulators/inhibitors that target a specific type of aggregate in the complex aggregation process.

## 2 RESULTS

### 2.1 Effect of betaine and proline on the kinetics of HI spherulites formation

**FIGURE 1:**
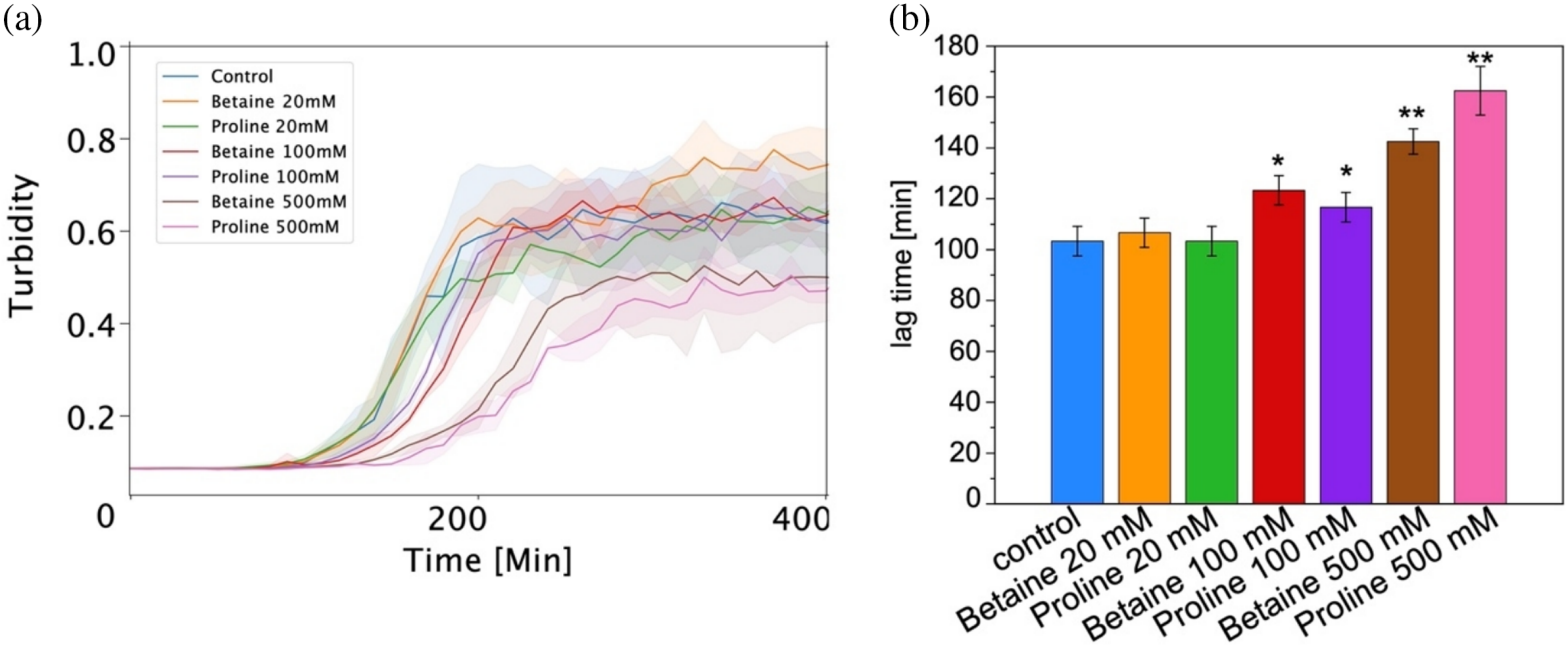
Bulk measurement of insulin aggregation at 45°C in the absence and presence of different concentrations of betaine or proline. (a) Turbidity kinetics of HI with different concentrations of betaine/proline. Each curve is the average of at least three replicates. The shaded lines present the standard deviation. (b) Distribution of the lag phase of insulin aggregation in different conditions. Error bars mean SD. Comparison of results in (b) was performed using two‐sided Welch *t*‐test to evaluate the differences of adding inhibitors to the control value.

We used an established protocol to induce HI aggregation to form spherulites (Foderà et al., 2010) and used betaine and proline to modulate insulin aggregation (Choudhary et al., 2015). We tested their modulatory effects at low (20 mM, insulin:ligand = 1:23), medium (100 mM, insulin:ligand = 1:116), and high (500 mM, insulin:ligand = 1:581) concentrations, respectively. Since some small ligands may bind to ThT and affect the accuracy of the ThT‐based fluorescence assay (Coelho‐Cerqueira et al., 2014; Hudson et al., 2009), and in our previous work, we have proved that turbidity and ThT‐based fluorescence provided identical results for insulin aggregation (Zhang et al., 2022). As a result, we used the turbidity signal to determine the ensemble aggregation kinetics (Figure 1a). As expected, the modulatory effect of betaine and proline on the formation of insulin spherulites at 45°C was enhanced with increasing concentrations (Figure 1). The lag phase of the aggregates with low concentration (20 mM) of betaine or proline are quite similar to the sample in the absence of betaine/proline (Figure 1b). The lag phase increased from ~103 ± 6 min (no ligands and low concentration ligands) to ~120 ± 6 min with 100 mM ligands, and ~160 ± 10 min with 500 mM ligands. It can be seen that the differences between no ligands, low concentration, and medium concentration ligands are very small and generally within the experimental error in the field. It seems that only the high concentration of betaine or proline shows a pronounced effect on the prolongation of the lag phase, indicating that the high concentration ligands could slow down the nucleation process. It is worth noting that even with similar insulin/ligand molar ratios, the effect of betaine or proline here is not so obvious compared to the work of Hosur and co‐workers (Choudhary et al., 2015), most likely due to different experimental conditions. Indeed, Choudhary et al. used a shaking condition at 250 rpm and an incubation temperature of 37°C to induce insulin fibrillation, whereas we used no shaking and a higher incubation temperature of 45°C to obtain insulin spherulites. The absence of shaking may affect the mixing between protein and ligand and, in turn, slow down the potential binding; and the higher incubation temperature could induce a faster nucleation and aggregation processes.

### 2.2 The final structures of HI aggregates formed in the presence of different concentrations of ligands

**FIGURE 2:**
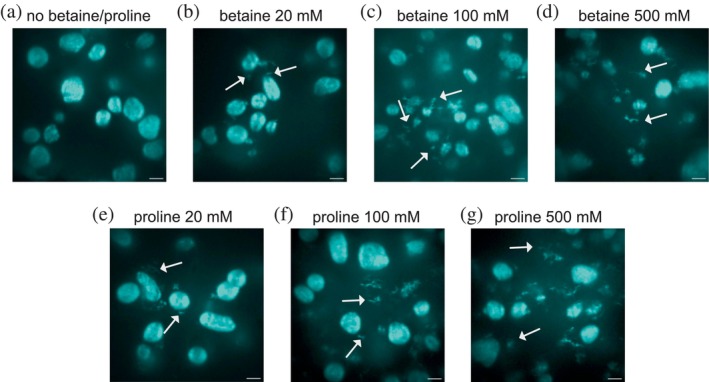
Spinning disk microscopy images of insulin aggregates grown at 45°C under different conditions: (a) no ligand; (b) with 20 mM betaine; (c) with 100 mM betaine; (d) with 500 mM betaine; (e) with 20 mM proline; (f) with 100 mM proline and (g) with 500 mM proline. The overall structure of the large aggregate appears not to be affected by the addition of ligands, albeit small (and more fibril‐like) aggregates are formed at high ligand concentrations. Write arrows indicate examples of small aggregates. Scale bars: 20 μm.

To study the final morphologies of insulin aggregates in the presence of different concentrations of the selected ligands after grown at 45°C for 24 h, we used spinning disk confocal microscopy (see Figure 2). Many spherulites were found in all the images of Figure 2, indicating that under this growth condition (low pH, high protein concentration and no shaking), most insulin aggregated into spherulites, which is consistent with our previous findings. Addition of ligands, especially at high ligand concentrations, decreased the absolute number of spherulites compared to those in the absence of ligands. Meanwhile, small aggregates covering an area of less than 10 μm^2^ were observed (some of the small aggregates are highlighted with white arrows in Figure 2), and the number fraction of small aggregates increased with ligand concentration. Table 1 shows the number of insulin spherulites and small aggregates observed within each condition, summed across four spinning disk microscopy images from four replicates for each condition. The corresponding numbers of spherulites and small aggregates in these four images under each condition are shown in Tables S1 and S2, respectively. It can be seen that in the absence of small ligands, >90% of insulin aggregated into spherulites. Interestingly, the impacts of betaine and proline are alike, yielding comparable quantities of spherulites and small aggregates at equivalent concentrations of either betaine or proline. Specifically, 5 small aggregates and 68 spherulites (~7% small aggregates) were observed from four spinning disk microscopy images in the absence of ligands. When 20 mM betaine or proline was added to the system, the number of small aggregates increased to ~16, with a percentage of ~27%. The number of small aggregates increases remarkably with increasing ligand concentration, with the corresponding percentage reaching 47% and >60% in the presence of 100 and 500 mM betaine or proline, respectively. This indicates that the addition of betaine or proline may shift the balance between the formation of big spherulite and the formation of small aggregates, thus reducing the average aggregate size. It is worth noting that a few small aggregates were also detected in the absence of ligands, further demonstrating that protein aggregation is a heterogeneous process. The addition of 20 mM ligand resulted in a notably greater number of small aggregates compared to the absence of ligands. If we consider the portion of protein in spherulites versus the one in small aggregates, the effect is so small that it cannot be reflected in the ensemble kinetics of insulin with no ligands and with 20 mM betaine/proline. This further demonstrates the necessity of studying the aggregation processes and morphology of individual aggregates to reveal the mechanism of small ligands.

**TABLE 1:** Number of spherulites and small aggregates formed in different conditions (the numbers are determined from four spinning disk microscopy images obtained from four replicates for each condition).

	No betaine/proline	Betaine 20 mM	Proline 20 mM	Betaine 100 mM	Proline 100 mM	Betaine 500 mM	Proline 500 mM
Spherulites	68	46	42	55	59	35	38
Small aggregates	5	17	16	49	52	62	58
Percentage of small aggregates	7%	27%	28%	47%	47%	64%	60%

### 2.3 Super resolution for direct observation of HI aggregation and deciphering the effect of small ligands

To directly observe the heterogeneous process in the absence and presence of these small ligands, we used our recently developed super‐resolution imaging method REPLOM (Zhang et al., 2022) that capitalizes on our single molecule readouts of stochastic processes (Bohr et al., 2019; Iversen et al., 2023; Jensen et al., 2021; Malle et al., 2022; Stella et al., 2018). We note that this process differs from the insulin oligomerization process (Bohr et al., 2023). According to the ensemble kinetics (Figure 1) and spinning disk images (Figure 2), we found that the effect of betaine and proline is quite similar, and their effects are more obvious at relatively high concentrations. As a result, we selected the medium concentration (100 mM proline) and the high concentration (500 mM betaine) to further investigate their effect on the growth of individual aggregates. REPLOM simultaneously records the spatially distinct binding events of multiple fluorescently labeled insulin molecules, enabling us to study the effect of the small ligands on the morphological development and growth pathway of each individual aggregate.

**FIGURE 3:**
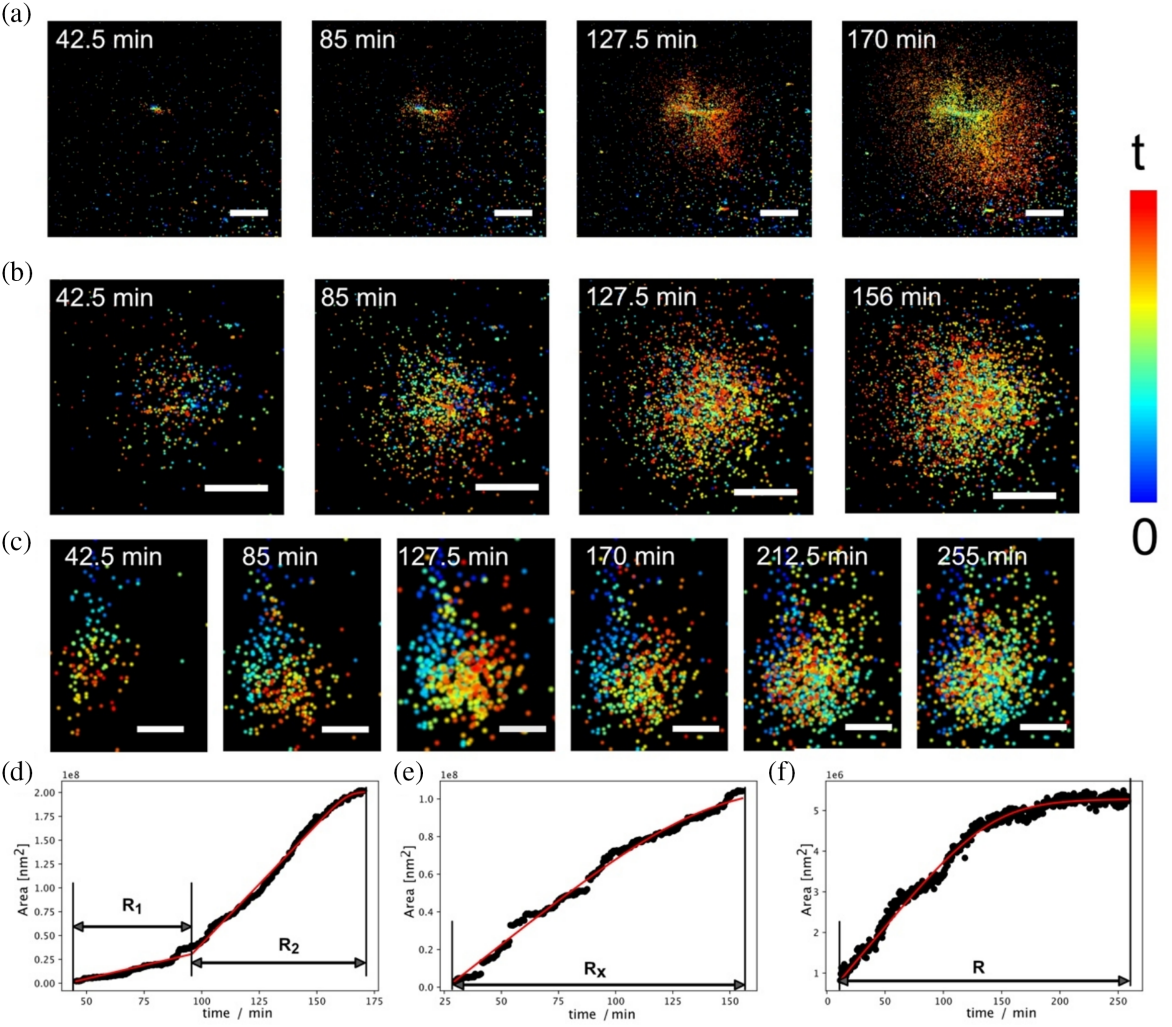
Direct recording of the modulation of insulin aggregation pathways by REPLOM: the anisotropic growth (a, d), the isotropic growth (b, e), and the small aggregate growth (c, f). (a–c) The temporal development of an anisotropic growth insulin spherulite (a), an isotropic growth insulin spherulite (b), and a small aggregate (c) observed by REPLOM. The reconstructed images with time series were plotted using ViSP software (Beheiry & Dahan, 2013). Scale bars: (a, b): 5 μm, (c): 1 μm. (d–f) The corresponding growth curves of the aggregate in a–c, respectively.

Individual aggregates were identified and quantified automatically by our SEMORE machine learning classification (Bender et al., 2024). We randomly examined 10, 7, and 18 fields of view for aggregation in the absence of ligands, in the presence of medium, and in the presence of high ligand concentrations, respectively. The scatter plot of the number of aggregates detected in each field of view is shown in Figure S1. The addition of small ligands resulted in a remarkable decrease in the number of aggregates in the fields of view (Figure S1). This may suggest that these ligands modulate the aggregation process or modulate their affinity to the positively charged poly‐L‐lysine surface, as the ligands are positively charged at low pH (Kalsoom et al., 2014). The direct morphology recordings showed that insulin spherulites can grow either anisotropically or isotropically in all three conditions (Figure 3a,b), which is consistent with our previously reported insulin spherulite growth pathways in the absence of ligands (Zhang et al., 2022; Zhou et al., 2023). In agreement with the spinning disk images, small aggregates were detected by REPLOM in the presence of betaine or proline. Figure 3c shows the growth of a small aggregate with 500 mM betaine in the system. See Videos [Link], [Link], [Link] for the real‐time growth of representative different types of aggregates in the presence of betaine/proline.

We further extracted the aggregation rates of each individual aggregate based on their real‐time morphology and area evolution observed by REPLOM. Figure 3a,b, individually show the morphological development over time of an anisotropic and an isotropic spherulite. For anisotropic growth, the corresponding rates *R*_1_ (dendritic fibril like structure) and *R*_2_ (branching part), and for isotropic growth, the rate *R*_*x*_, are shown in Figure 3d,e, respectively. In contrast to the spherulites, the morphology of the small aggregates changes very little after ~127 min (Figure 3c), with only a few insulin monomers subsequently bound. The final coverage area is ~20 times smaller than that of the anisotropic and isotropic spherulites (see Figure 3f). This indicates that the aggregation of small aggregates was terminated by the presence of 500 mM betaine.

**FIGURE 4:**
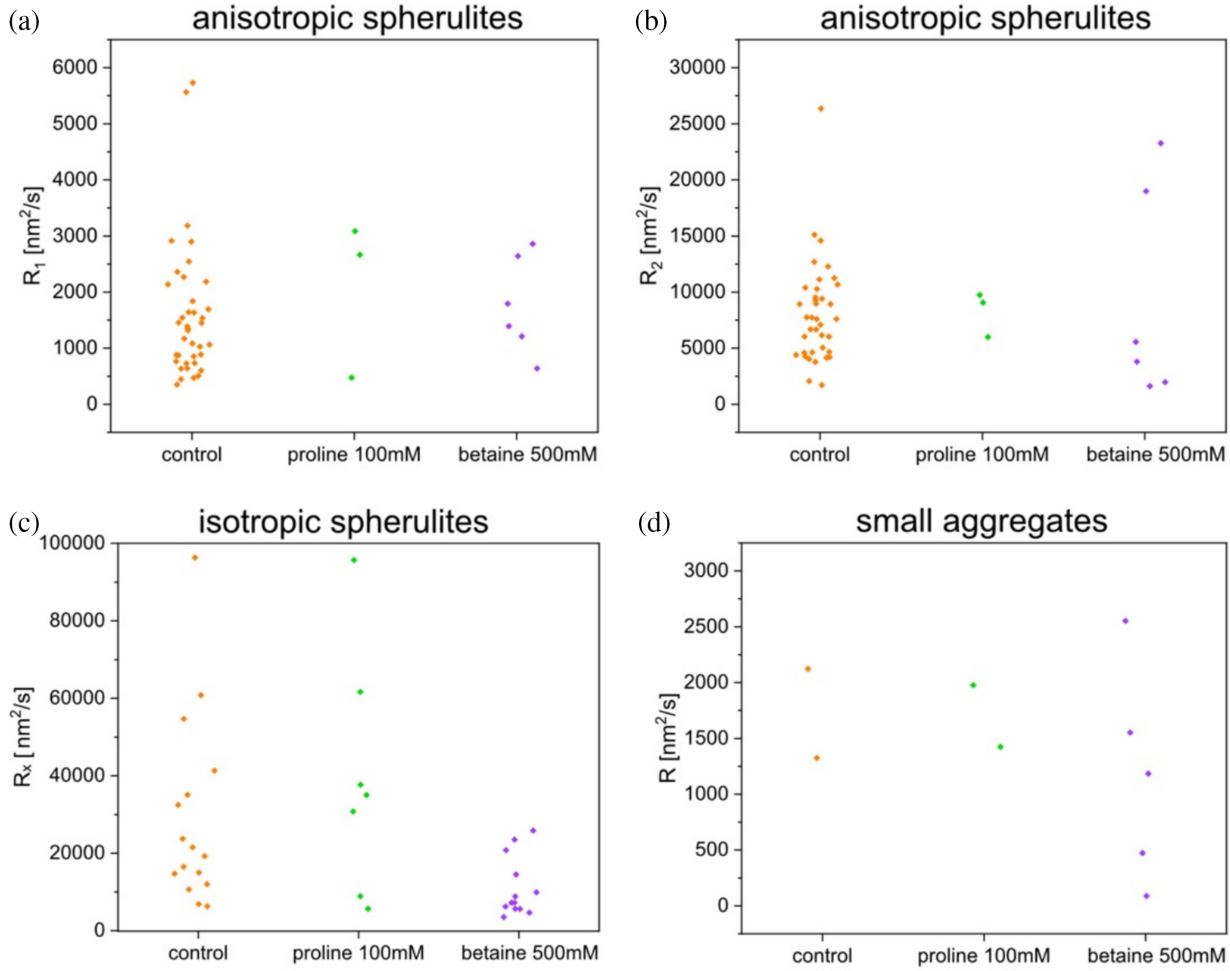
The real‐time growth rates of each REPLOM‐observed aggregate as a function of ligand concentrations. (a) *R*_1_ of anisotropic spherulites, (b) *R*_2_ of anisotropic spherulites, (c) *R*_*x*_ of isotropic spherulites, and (d) *R* of small aggregates. The aggregates are detected from 10, 7, and 18 fields of view for aggregation in the absence of ligands, in the presence of 100 mM proline, and in the presence of 500 mM betaine, respectively.

Figure 4 shows the growth rates of each aggregate observed by REPLOM under different conditions. The corresponding number and average rates of each type of aggregate are shown in Table 2. As expected, small aggregates grew much slower than insulin spherulites. It is noteworthy that the number of aggregates detected in the presence of 100 mM proline or 500 mM betaine is much lower than that in the absence of ligands. This may be because both the ligands and the poly‐L‐lysine covered surface are positively charged, confirming that betaine/proline binds to the aggregates and prevents them from docking to the positively charged surface. The growth rates for the same type of aggregates under no ligands and with 100 mM proline have no significant difference, while it appears that the rates of isotropic spherulites and small aggregates in the presence of 500 mM betaine are slightly slower than under the other two conditions. As described in our previous work (Zhou et al., 2023), the effect of small ligands on protein aggregation depends not only on the amount of ligand and protein molecules but also on the available binding sites and possible differences in hydrophobicity or structure of these binding sites. In this work, the amount of 100 mM proline or the binding sites on anisotropic spherulites may not be sufficient for the ligands to affect the aggregation rates of spherulites. The addition of high concentration ligands (e.g., 500 mM betaine) not only causes the formation of more small aggregates but also reduces the rates of isotropic spherulites, indicating that isotropic spherulites may have more binding sites than anisotropic spherulites.

**TABLE 2:** Number of different types of aggregates detected by REPLOM and the average rates in different conditions.

	Control		Proline 100 mM		Betaine 500 mM	
	Number	Average rates (nm^2^/s)	Number	Average rates (nm^2^/s)	Number	Average rates (nm^2^/s)
Anisotropic spherulite_*R*_1_	39	1599 ± 193	3	2074 ± 809	6	1756 ± 350
Anisotropic spherulite_*R*_2_	39	8073 ± 726	3	8266 ± 1149	6	9205 ± 3855
Isotropic spherulite_*R*_*x*_	16	29,211 ± 6044	7	39,347 ± 11,773	13	11,054 ± 2111
Small aggregate_*R*	2	1724 ± 399	2	1700 ± 276	5	1170 ± 431

*Note*: Error bar means SE.

## 3 DISCUSSION

**FIGURE 5:**
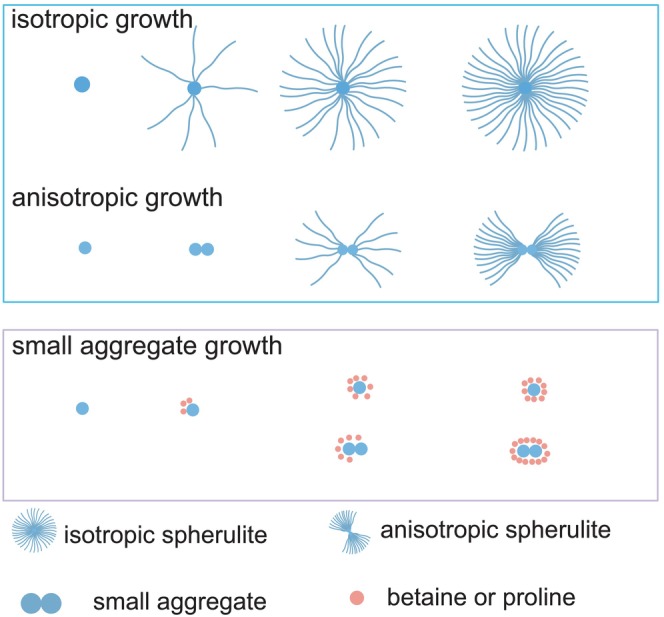
Illustration of the effect of betaine or proline on insulin aggregation. Please note that this scheme is not to scale.

We recorded the overall effects of betaine and proline by the ensemble aggregation kinetics and detected the final morphologies of the obtained insulin aggregates by spinning disk confocal microscopy. Interestingly, our approach allowed us to report that the addition of betaine or proline resulted in the formation of more small aggregates, while the sizes and morphologies of the big spherulites showed no apparent difference from those grown without ligands (Figure 2). This effect would be detected by imaging methodologies, such as fluorescent microscopy, EM, or AFM, but would be masked by the ensemble aggregation kinetic results. Then we used the advanced method REPLOM to directly observe the aggregation pathways of each individual aggregate and extracted their aggregation rates and abundances at different concentrations of small ligands. Samuel et al. pointed out that small ligands like proline could bind to folding intermediates and modulate protein aggregation (Samuel et al., 2000). After careful inspection of the growth pathways and aggregation rates of each individual aggregate in the three conditions, we argue that the formation of more small aggregates in the presence of ligands could be due to betaine and proline binding to the partially folded aggregates/oligomers, thus slowing down their aggregation rates. When an aggregate is wrapped by the ligands, it cannot recruit more protein monomers, thereby resulting in its aggregation being terminated and the formation of small aggregates (Figure 5). Considering the size difference between the aggregates and the ligands, one should expect a large number of ligands to sequester individual aggregates; this may explain why ligands like betaine and proline only have good modulation effects at relatively high concentrations, and why we observed more small aggregates with increasing ligand concentrations. Interestingly, even at high concentrations, the large portion of spherulites continues to grow in an anisotropic or isotropic way, just as in the absence of small ligands, indicating that the amount of ligands or the interactions between ligands and spherulites may not be sufficient to affect spherulite aggregation. The mechanism of this all‐or‐nothing response is unclear and needs further investigation.

Our combined results (spinning disk and REPLOM) revealed how small ligands affect the morphology and dynamics of each of the aggregation subsets. These findings demonstrate the highly heterogeneous nature of the process and highlight that understanding how the small ligands affect the aggregation pathways at the single aggregate level is crucial for deciphering their mechanism. Advanced single‐molecule imaging methods such as REPLOM could be a powerful approach to study the modulatory effects of small ligands at the single aggregate level, which can be used as a promising platform for the development of next‐generation small molecule therapeutics for neurodegenerative diseases.

## 4 MATERIALS AND METHODS

### 4.1 Materials

Chemicals were of analytical grade and purchased from Sigma‐Aldrich (Denmark) unless otherwise stated. Alexa Fluor 647 NHS Ester was acquired from ThermoFisher Scientific.

### 4.2 Human insulin labeling

Human insulin (HI) labeling followed the protocol of our previous work (Zhang et al., 2022). In detail, 5 μL anhydrous DMSO dissolved Alexa Fluor 647‐NHS Ester solution with a concentration of 2 mg/mL was mixed with 1 mL 5 mg/mL HI monomer solution at pH 8.3. The mixture was allowed to stand at room temperature for 2 h to complete the conjugation. Then the excess of free dyes was removed by a PD SpinTrap G‐25 column (GE Healthcare). The purified Alexa Fluor 647 labeled insulin was divided into aliquots and stored at −80°C.

### 4.3 The preparation of HI spherulites

HI powder was dissolved in pH ~ 1.7 containing 0.5 M NaCl, 20% acetic acid with a concentration of 5 mg/mL. To perform REPLOM, Alexa Fluor 647 labeled HI was mixed with the unlabeled HI monomer with a ratio of around 1 to 10,000 (labeled to unlabeled). Betaine or proline was added to the HI solution to reach a high (500 mM), a medium (100 mM) or a low (20 mM) concentration. Then the solution was filtered through 0.22 μm filters (LABSOLUTE) and incubated at 45°C without shaking.

### 4.4 Turbidity‐based kinetics assay

For in situ turbidity‐based kinetics assay, the aggregation reaction was carried out in a plate‐reader system (iD3, Molecule Device) with a 96‐well sealed plate (Nalge Nunc, ThermoFisher Scientific). Each well contained 5 mg/mL HI solution, and the absence or presence of betaine/proline at a low, medium, or high concentration in a total volume of 200 μL. The plates were covered with a self‐adhesive sealing film (nerbe plus) to avoid evaporation of the samples and incubated at 45°C without mechanical shaking. The turbidity signal was detected every 10 min by exciting the samples at a 450 nm wavelength. A minimum of three plate replicates were made for each condition.

### 4.5 Spinning disk confocal microscopy

We used a SpinSR 10‐spinning disk confocal super‐resolution microscope (Olympus) to observe the final morphologies of HI aggregates with/without ligands. The insulin aggregates were stained by Thioflavin T (ThT) and excited with a 488 nm laser (OBIS COHERENT).

### 4.6 Real‐time kinetic via binding and photobleaching localization microscopy

REPLOM was performed on an inverted total internal reflection fluorescence (TIRF) microscope (Olympus IX‐83) with a temperature adjustable 100x oil immersion objective (UAPON 100XOTIRF, NA = 1.49, Olympus). Generally, the solutions containing 5 mg/mL HI monomer in the presence or absence of ligands were first incubated in a block heater at 45°C for ~7–16 h to skip the lag phase (the length of pre‐incubation time depends on the concentration of small ligands in the solution). Then the sample was transferred to poly‐L‐lysine coated glass slide chambers (Chen et al., 2017) and covered by a lip to prevent buffer evaporation. A heating unit 2000 (PECON) was used to keep the incubation temperature at 45°C during the imaging process. Alexa Fluor 647 labeled HI was excited by a 640 nm solid state laser line (Olympus) and reflected to a quad band filter cube (dichroic mirrors ZT640rdc, ZT488rdc and ZT532rdc for splitting and single‐band pass filters FF02‐482/18‐25, FF01‐532/3‐25 and FF01‐640/14‐25). The signal was detected by an EMCCD camera (imagEM X2, Hamamatsu). Imaging was performed with an exposure time of 30 ms followed by a waiting time for each frame of 30 s to capture the aggregation processes in real time.

### 4.7 Quantification of growth kinetics observed by REPLOM

The REPLOM data were first analyzed using ThunderSTORM (Ovesný et al., 2014). We set an intensity threshold to remove the possible false positives or poor‐quality detections. Drift correction was done using NanoJ‐Core (Laine et al., 2019). Individual protein aggregates were extracted by SEMORE, a semi‐automatic unsupervised clustering pipeline that utilizes an incremental clustering methodology and in‐depth automatic analysis based on the structural densities and temporal pathways to cumulatively dissect and segment both individual and intertwined structures (Bender et al., 2024). SEMORE has been validated through qualitative analyses of both dynamic and static SMLM datasets, including time‐resolved insulin aggregates, dynamic live‐cell PALM of ryanodine receptors, and dSTORM imaging of nuclear pore complexes, broblast growth receptor 1, and sptPALM of Syntaxin 1a. In particular, the quantitative performance of the method was evaluated on simulated data representing three biologically relevant dynamic structures, with an accuracy of >90%.

## AUTHOR CONTRIBUTIONS

**Steen W. B. Bender:** Software; data curation; formal analysis; investigation; writing – review and editing. **Jacob Kæstel‐Hansen:** Data curation; formal analysis; validation; writing – review and editing; software. **Vito Foderà:** Supervision; writing – review and editing; funding acquisition. **Nikos S. Hatzakis:** Supervision; funding acquisition; writing – review and editing; project administration; methodology. **Min Zhang:** Methodology; investigation; funding acquisition; supervision; writing – original draft; writing – review and editing; formal analysis; project administration.

## Supporting information


**VIDEO S1:** Real time growth of an anisotropic spherulite with 100 mM proline.

**VIDEO S2:** Real time growth of an isotropic spherulite with 500 mM betaine.

**VIDEO S3:** Real time growth of a small aggregate with 500 mM betaine.

**FIGURE S1.** Number of the detected insulin aggregates in each field of view per condition.
**TABLE S1:** Number of spherulites detected in each spinning disk microscopy image used for the statistics in Table 1. Error bar means SE.
**TABLE S2:** Number of small aggregates detected in each spinning disk microscopy image used for the statistics in Table 1. Error bar means SE.

## Data Availability

The data that support the findings of this study are available from the corresponding author upon reasonable request.
