# Supplementary material for: Modulation of insulin aggregation by betaine and proline directly observed via real‐time super‐resolution microscopy
Source: Protein Sci. 2025 May 15;34(6):e70149. doi: 10.1002/pro.70149 (PMC12079458; doi:10.1002/pro.70149)
Supplement: Supplementary file 4 — FIGURE S1. Number of the detected insulin aggregates in each field of view per condition. TABLE S1: Number of spherulites detected in each spinning disk microscopy image used for the statistics in Table 1. Error bar means SE. TABLE S2: Number of small aggregates detected in each spinning disk microscopy image used for the statistics in Table 1. Error bar means SE. [file PRO-34-e70149-s003.docx]

**Inhibition of insulin spherulite formation by betaine and proline directly observed via real-time super-resolution microscopy**

*Steen W. B. Bender ^1,2^, Jacob Kæstel-Hansen ^1,2,3^, Vito Foderà ^4^,  Nikos S. Hatzakis*^1,2,3^and Min Zhang *^1,2,3^*

^1^ Department of Chemistry, University of Copenhagen, Thorvaldsensvej 40, DK-1871 Copenhagen, Denmark.

^2^ Center for Optimized Oligo Escape and Control of Disease, University of Copenhagen, Thorvaldsensvej 40, DK-1871 Copenhagen, Denmark.

^3^ Center for 4D cellular dynamics, University of Copenhagen, Thorvaldsensvej 40, DK-1871 Copenhagen, Denmark.

^4^ Department of Pharmacy, University of Copenhagen, Universitetsparken 2, DK-2100 Copenhagen, Denmark.

**Corresponding Author**

*Correspondence should be addressed to:

Nikos S. Hatzakis: [hatzakis@chem.ku.dk](mailto:hatzakis@chem.ku.dk), and Min Zhang: [min.zhang@chem.ku.dk](mailto:min.zhang@chem.ku.dk)

**Figure S1.** Number of the detected insulin aggregates in each field of view per condition.

**Table S1.** Number of spherulites detected in each spinning disk microscopy image used for the statistics in Table 1. Error bar means SE.

| image | No betaine/proline | Betaine 20mM | Proline 20mM | Betaine 100mM | Proline 100mM | Betaine 500mM | Proline 500mM |
| --- | --- | --- | --- | --- | --- | --- | --- |
| 1 | 17 | 15 | 11 | 18 | 11 | 9 | 9 |
| 2 | 13 | 9 | 9 | 13 | 13 | 11 | 11 |
| 3 | 19 | 9 | 8 | 10 | 17 | 6 | 11 |
| 4 | 19 | 13 | 14 | 14 | 18 | 9 | 7 |
| average | 17±1.4 | 11.5±1.5 | 10.5±1.3 | 13.8±1.7 | 14.8±1.7 | 8.8±1.0 | 9.5±1.0 |

**Table S2.** Number of small aggregates detected in each spinning disk microscopy image used for the statistics in Table 1. Error bar means SE.

| image | No betaine/proline | Betaine 20mM | Proline 20mM | Betaine 100mM | Proline 100mM | Betaine 500mM | Proline 500mM |
| --- | --- | --- | --- | --- | --- | --- | --- |
| 1 | 1 | 5 | 7 | 12 | 12 | 12 | 20 |
| 2 | 2 | 3 | 3 | 8 | 14 | 20 | 12 |
| 3 | 2 | 7 | 4 | 8 | 18 | 17 | 16 |
| 4 | 0 | 2 | 2 | 21 | 8 | 13 | 10 |
| average | 1.3±0.5 | 4.3±1.1 | 4±1.1 | 12.3±3.1 | 13±2.1 | 15.5±1.8 | 14.5±2.2 |
